# CanvasDB: a local database infrastructure for analysis of targeted- and whole genome re-sequencing projects

**DOI:** 10.1093/database/bau098

**Published:** 2014-10-03

**Authors:** Adam Ameur, Ignas Bunikis, Stefan Enroth, Ulf Gyllensten

**Affiliations:** Department of Immunology, Genetics and Pathology, Science for Life Laboratory, Uppsala University, Sweden

## Abstract

*CanvasDB* is an infrastructure for management and analysis of genetic variants from massively parallel sequencing (MPS) projects. The system stores SNP and indel calls in a local database, designed to handle very large datasets, to allow for rapid analysis using simple commands in R. Functional annotations are included in the system, making it suitable for direct identification of disease-causing mutations in human exome- (WES) or whole-genome sequencing (WGS) projects. The system has a built-in filtering function implemented to simultaneously take into account variant calls from all individual samples. This enables advanced comparative analysis of variant distribution between groups of samples, including detection of candidate causative mutations within family structures and genome-wide association by sequencing. In most cases, these analyses are executed within just a matter of seconds, even when there are several hundreds of samples and millions of variants in the database. We demonstrate the scalability of *canvasDB* by importing the individual variant calls from all 1092 individuals present in the 1000 Genomes Project into the system, over 4.4 billion SNPs and indels in total. Our results show that *canvasDB* makes it possible to perform advanced analyses of large-scale WGS projects on a local server.

**Database URL:** https://github.com/UppsalaGenomeCenter/CanvasDB

## Introduction

As the capacity and accuracy of massively parallel sequencing (MPS) technologies continue to improve, they are becoming an affordable alternative to other molecular genetics techniques for a wide range of studies in human and medical genetics. Already today, high-throughput DNA sequencing is an established method to screen for disease-causing mutations (1, 2). Increasingly, MPS is also being used to investigate the genetic variation in different population cohorts (3–6) and to gain knowledge of our evolutionary history (7–9). With all these new and exciting possibilities, the number of human DNA sequences that will become available is likely to grow dramatically over the coming years. Moreover, the steadily decreasing cost per sample for DNA sequencing will make it possible also for smaller institutions and research groups to produce sequence data for large numbers of individuals.

High-throughput DNA sequencing can be performed either by generating data for the entire genome [whole-genome sequencing (WGS)], or by selectively targeting the regions of interest. At present, whole-exome sequencing (WES) is routinely being used to investigate the protein coding part of the human genome. The main benefit of WES is that many samples can be multiplexed in a single sequencing run, thereby reducing the cost. However, the methods for capturing the exome are not perfect, and some parts of the coding sequences are typically missed in WES. One way around this problem is to instead perform WGS, which has the additional benefit that it yields much more information about the genome compared with targeted approaches (10). WGS studies are likely to become more common in the future given that the throughput of the MPS instruments continue to increase, thereby driving down the cost per base of sequencing.

There is a need for development of streamlined bioinformatics analysis pipelines to keep up with the production rate of the DNA sequencers. Many human re-sequencing projects are in the scale of several hundreds or thousands of samples (4, 9, 11), and management of the data and extraction of the genetic variants of interest from such datasets can be a major challenge. Analysis of genetic variants from high-throughput experiments typically involves a number of different filtering strategies. Many of the filtering tasks are straightforward, while others are more complicated. A relatively uncomplicated type of filtering is to detect all SNPs and indels that are present only in one or a few individuals, but absent from the population as a whole. This type of filtering is common when searching for rare disease-causing mutations (see (12) and references within). An example of a more complex analysis is to identify variants that occur with a high frequency in a large group of samples, while it is present at a low frequency in another group. Such group-based filtering analyses are fundamental for many types of studies, such as when screening for genetic differences between population cohorts or doing sequence-based genome-wide association studies (SEQ-GWAS).

The typical approach for filtering of SNP and indel data is to start from a file containing all variants detected by sequencing and to apply successive filters, thereby reducing the original list of variants into a manageable subset of candidate variants. These filters are usually applied to remove false positive variants, common SNPs/indels from databases like dbSNP (13), and variants having no effect on the amino acid sequence. Although such filtering strategies might successfully pin down the disease-causing variant, the analysis workflow is far from optimal. Each filtering step takes time and usually produces temporary files to be used as input to the subsequent step. This implies that the analysis process becomes cumbersome and possibly error-prone, at least for larger projects or when some of the filtering steps need to be rerun using different parameters. Another drawback is that the approach with subsequent filtering steps is not suitable for more complex types of analyses, such as filtering based on differences in allele frequencies between groups of samples (e.g. between cases and controls in an association study of a complex disease).

Here we present *canvasDB* (CANdidate Variant Analysis System and Data Base), a database-oriented system that allows for efficient management and analysis of genetic variants from high-throughput sequencing projects using R (14). The system can be installed on a local computer or server, and does not rely on an external computer or ‘cloud’, which means that all generated genetic data stays in-house and does not need to be transferred to an outside location.

## Methods

### Database structure

The *canvasDB* MySQL database contains tables for sample information, run information, SNPs, indels and variant annotations. SNPs and indels are loaded into distinct tables for each sample, where information about homo- or heterozygozity, read depth for the alleles and quality of variant calls are stored. A fundamental part of the system is that we have constructed SNP and indel summary tables to allow for extremely rapid filtering of variants between groups of samples. These two tables hold information about all the variants that have been loaded into the system: chromosome, position, alternative allele, the samples in which the variant is found, dbSNP rs-id (if any), functional effect of variant (nonsynonymous, frameshift, splicing etc), and various scores for conservation and predicted consequence on protein function. Having all this information stored in two indexed MySQL tables enables much of the filtering analyses to be performed by simple table lookups in MySQL, which are executed very rapidly. It is crucial that summary tables are updated whenever new variants are added or removed from the database, and this is ensured by our built-in R functions for adding and removing samples.

### File formats for variant call data

C*anvasDB* system has an internal format for variant calls, but can also handle other common file formats, like .vcf, .txt and .gff files. The system is platform independent, and in our current installation, we have stored data from the SOLiD, Illumina and Ion Proton systems. All variant call data are first parsed into the internal *canvasDB* format. This makes it easy to incorporate new file formats into the system by writing functions that converts the input file into a *canvasDB* formatted file. For a more detailed description of the file format, see supplementary information.

### Adding new samples

The easiest way to add new samples into *canvasDB* is to construct a text file from a predefined template (see supplementary information), where each row corresponds to a sample. Each row in this file contains some brief information about the sample and how the sequencing experiment was carried out, and also file paths to the SNP and indel raw data files. By calling a batch import function from R, all samples in the text file are imported into *canvasDB*. If any of the samples already exist in the database (i.e. has an identical sample id), they are not imported.

When new data are imported, all SNPs and indels that are being added into the system are first annotated with information from refSeq (15) and dbSNP (13), as well as SIFT (16), Polyphen2 (17), PhyloP (18), LRT (19), MutationTaster (20) and GERP (21) from dbNSFP (22). In our current configuration, RefSeq annotations are performed by the ANNOVAR software (23). All other SNP and indel annotations are taken from pre-installed annotation tables in the database. The annotation is only performed once for each variant that is added to the system, and the resulting information is stored in the SNP and indel summary tables. In this way, we can rapidly populate the database with large quantities of data. On our local server (see hardware specifications below), it takes about three hours to populate the database with 428 WES samples (27.6 million variants in total). The entire 1000 Genomes data (1092 samples, 4.44 billion variants) was annotated and imported in <96 h.

### Writing analysis functions

Communication between the database and R is done through RMySQL(cran.r-project.org/web/packages/RMySQL/ index.html). Any custom MySQL queries can be executed in this way, and the results are returned into R. We have implemented R functions for some specific tasks, i.e. performing filtering between samples, populating the database with sample and variant data and generating statistics. These built-in functions have been optimized for so that complex analyses can rapidly be performed on large datasets. However, there is also a possibility to add new R functions and thereby develop new types of analyses of the data in the database. Because the data are directly returned into R, all packages and functions in Bioconductor (24) can be integrated with the system.

### Evaluating filtering performance of canvasDB

For evaluation, we implemented a naïve filtering analysis function and compared the performance with our more advanced strategy where SNP and indel summary tables are used. We then tested both methods in a situation where the task was to detect all variants that are common between two individuals in the WES database, while absent from all other samples. In the naïve approach, we simply joined the two database tables holding variants from two individuals to identify all shared variants. We then looped through all remaining samples in the database to exclude any of these shared variants that were also detected in any of the other samples. All these tests were performed using a newly installed database and using the ‘SQL_NO_CACHE’ option. This implies that the MySQL cache, which could potentially speed up some of these queries, was not used in these evaluations.

### Installation for exome-seq data

Our local installation for WES data used in this manuscript consisted of 428 exomes sequenced on the SOLiD and Ion Proton systems. In total there were 26 428 294 SNPs and 1 164 644 indels in the database and new datasets are continuously being added. There is a mix of samples in the database, with some individuals being affected by various diseases, while others are healthy controls.

### Installation for the 1000 Genomes Project

To set up a *canvasDB* installation consisting of variants from the 1000 Genomes Project (11), compressed VCF-files containing all individual genotypes split on chromosomes was downloaded from the 1000Genomes ftp site (ftp://ftp.1000genomes.ebi.ac.uk/vol1/ftp/release/20110521/). These VCF files contain SNPs and indels for 1092 individuals from 14 different populations; ASW (n = 61), CEU (n = 85), CHB (n = 97), CHS (n = 100), CLM (n = 60), FIN (n = 93), GBR (n = 89), IBS (n = 14), JPT (n = 89), LWK (n = 97), MXL (n = 66), PUR (n = 55), TSI (n = 98) and YRI (n = 88). From the VCF files we then constructed 1092 SNP files and 1092 indel files, one for each individual, and loaded all data into inhouseDB. In total, there are 4 041 538 891 SNPs and 399 636 723 indels in this installation, and the size of the whole database is 354 GB.

### Hardware and software

The canvasDB installations and analyses in this manuscript were performed on an Ubuntu 12.04 (precise) 64-bit system, with 12 CPUs and 36 Gb RAM, and MySQL version 5.5.29 and R version 3.0.0.

## Results

### Overview of the canvasDB system

**Figure 1.**
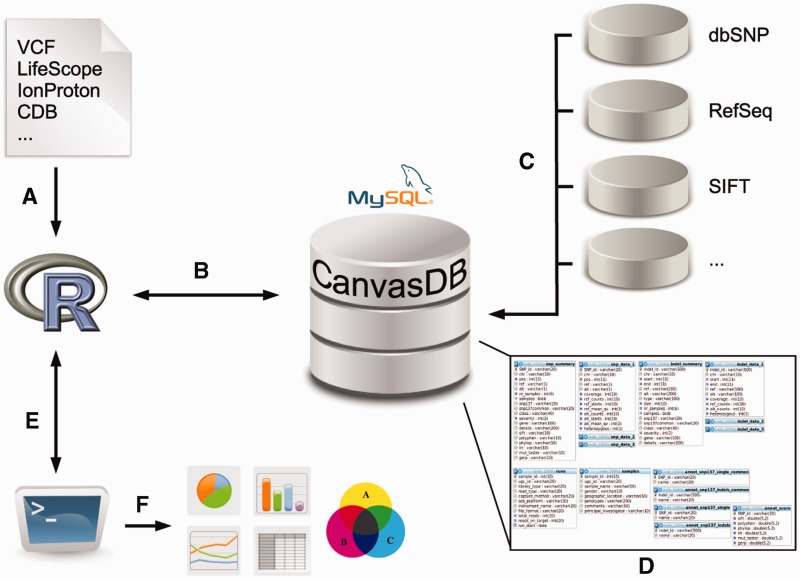
Overview of the canvasDB system. The figure shows a schematic view of the workflow how variant data are imported, stored and analyzed within the canvasDB system. (**A**, **B**) Variant calls for SNP are added to the system using a function call in R; different file formats are supported. (**C**) All new variants that are not already stored within the system are annotated against databases like dbSNP, RefSeq, SIFT, etc. (**D**) The variant data, annotations and information about the samples are stored in MySQL database tables, in a way that allows for rapid comparative analyses of variants between samples. (**E**) Analysis on the variants within the canvasDB system is performed through functions in R, using the RMySQL package. (**F**) Using pre-defined or custom analysis functions in R/Bioconductor, it is possible to generate lists of candidate disease-causing mutations, or any other types of analysis results, statistics or graphical plots based on the variant data in the database.

The *canvasDB* system consists of a MySQL database, which is installed on a local computer or server and accessed through an interface in R (14). The database can store all SNP and indel calls from thousands of samples, along with functional annotation of the variants and, optionally, additional information about samples and sequencing runs. An overview of the system is shown in Figure 1; see Methods for more details.

The database has been designed in such a way that it allows for an extremely rapid filtering of variants between groups of samples. A fundamental part of this design is the SNP and indel summary tables that store information about all variants imported into the system and keep track of all samples in which they have been detected. Using this approach we are able to store all relevant information for filtering in two large MySQL tables, which are indexed by the database engine for efficient searches. Moreover, R functions and MySQL queries have been optimized to enable fast insert of data and rapid filtering analyses. Compared with naïve analysis approaches, our solution can reduce the execution time of different filterings by several orders of magnitude. More details about the summary table database structures and how the system makes use of these tables for rapid variant filtering are given in Supplementary Tables S1 and S2 and in a separate section of the supplementary information.

To set up a new installation of *canvasDB*, a new empty database is created on a local server. The database is then ready to be populated with SNP and indel data, and this is done through a function call from R. The system has an internal file format for variant calls, but other common file types (e.g. VCF) are also supported. Multiple samples can be imported into the system in a batch mode and variants are automatically annotated with rs-ids from dbSNP (13), effect of variants on refSeq genes (15) and with various scores for conservation and predicted consequences on protein function through dbNSFP (22). In our current installation, we use ANNOVAR (23) along with hg19 annotation tables (downloaded from ANNOVAR) for refSeq annotations, while all the other annotations are done through local database tables. When the samples have been imported into *canvasDB* the data can be analyzed using simple commands in R.

### CanvasDB is suitable both for WES and WGS

**Figure 2.**
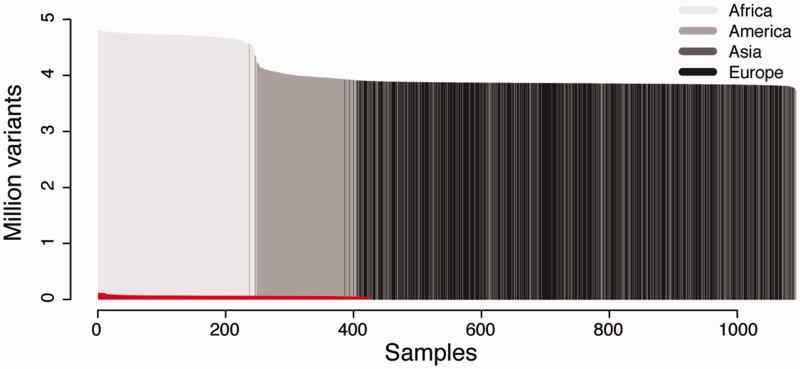
Datasets used for testing the performance of *canvasDB*. The figure shows the number of individuals (x-axis) and number of variants (y-axis) in the 1000 Genomes data and 428 locally produced WES samples. The 1000 Genomes samples are colored in different shades of gray for populations on different continents. The WES samples are colored in red. All samples have been ordered for each of the datasets with the individuals having the highest number of variants furthest to the left.

We created two separate installations of *canvasDB* to test the performance in different situations (see Figure 2). The first installation contains the results from 428 locally sequenced WES samples (see Methods), while the other contains all SNP and indel variants detected from the pilot phase of the 1000 Genomes Project (11). As shown in Figure 2, the 1000 Genomes dataset is extremely large, and to our knowledge it is the largest publicly available collection of SNPs and indels from whole human genome sequencing (WGS). Although the ∼28 million variants from the 428 samples in our WES database is also a very large dataset, it is barely visible when compared with the 1000 Genomes data, which includes >4.4 billion variants from 1092 individuals (see Figure 2). We therefore consider the 1000 Genomes data as an ideal test data set to evaluate the scalability and performance of *canvasDB* for storage and analysis of the data from large-scale WGS projects. The whole process of importing the 1000 Genomes data into the system, including functional annotations of all variants, was completed within four days.

### CanvasDB has a powerful filtering tool

**Figure 3.**
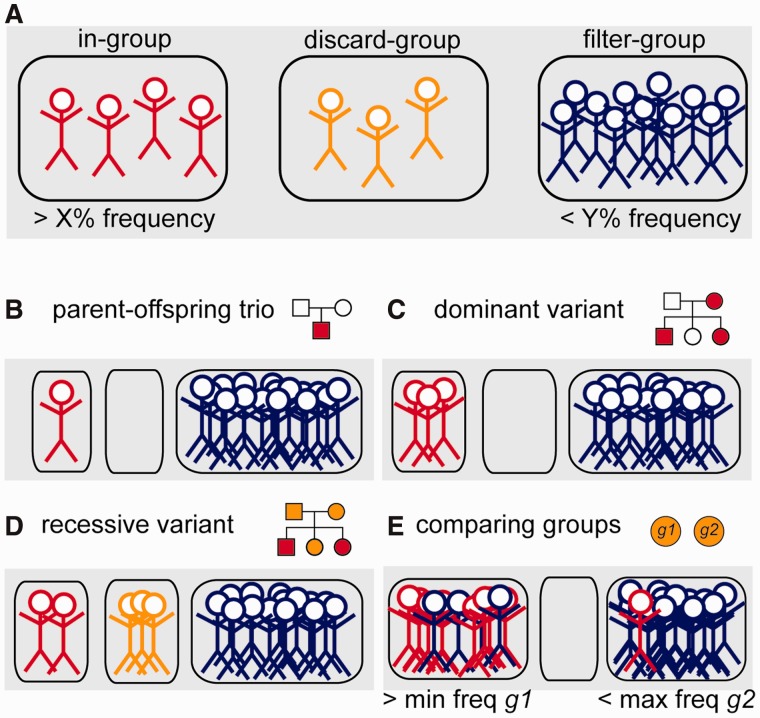
Schematic representation of the variant filtering function. (**A**) All individuals in the database are divided into three distinct groups; the ‘in-group’ (to the left, in red), ‘discard-group’ (in the middle, orange) and ‘filter-group’ (to the right, blue). The function then returns all SNPs or indels in the system that are detected in at least X% of the ‘in-group’ and at the same time at most Y% of the ‘filter-group’. Individuals in the ‘discard-group’ are excluded from the analysis. With this filtering function, it is possible to perform many different types of filtering, as shown in the examples below. (**B**) Filtering to detect a de novo mutation in a child of a sequenced mother–father–child trio. (**C**) Detection of a dominant variant in a family. (**D**) Detection of a recessive variant in a family. In this case, family members that may be healthy carriers are put in the ‘discard-group’. (**E**) Detection of variants that occur with frequency of at least X% in one group of samples (‘in-group’, *g1*) and at least Y% in all other samples (‘filter-group’, *g2*).

We designed the *canvasDB* system to efficiently execute all types of filtering tasks. The filtering is done by a function in R, which extracts data directly from the SNP and indel summary tables. For most filtering tasks, the execution takes only a few seconds, even when there are several hundreds of samples in the system and millions of variants in the summary tables. To make the filtering flexible, the user divides the samples into three distinct groups, named ‘in-’, ‘discard-’ and ‘filter-’ groups (see Figure 3A). The ‘in-group’ contains the individuals among which we are looking for a shared variant. The ‘filter-group’ can be seen as negative control samples, i.e. those where the same variant should not occur. The ‘discard-group’ contains such samples that are not included in the analysis.

With this grouping of samples we can perform filtering analyses for many different purposes, as illustrated by the examples in Figure 3B–E. By having a single individual in the ‘in-group’ and all others in the ‘filter-group’, we can identify variants that are unique for that individual. This approach is useful when screening for *de novo* mutations occurring in a child of a sequenced mother-father-child trio (Figure 3B). Because the ‘filter-group’ consists of all other samples including the parents, the filtering at the same time removes inherited variants from the parents and false positives (due to the sequencing technology) that appear in multiple samples. Also, in the filtering we can directly select for nonsynonymous, stop-gain or splice-site mutations that are not present in dbSNP (or dbSNPcommon), thereby reducing the list of candidate variants even further. Another filtering scenario is to detect disease-causing variants among members of a family, where some individuals are affected while others may be healthy carriers. In the examples in Figure 3C and D, we have all the affected individuals in the ‘in-group’ and all unrelated samples in the ‘filter-group’. The related individuals who could be healthy carriers are placed in the ‘discard-group’, as we are uncertain whether or not they carry the mutation, and they would influence the filtering results if included in any of the other groups.

A more complex type of analysis is to detect variants that differ in allele frequencies between groups of samples. Such filterings are useful when searching for genetic variants that are more common in one population compared with another, or in a group of patients compared to healthy controls. In Figure 3D we show an example where two groups of samples, *g1* and *g2*, are compared. The filtering function allows us to screen for variants that occur with high frequency among individuals in *g1*, while at the same time having a low frequency in *g2*. In practice, we can do this by assigning the ‘in-group’ as *g1* and the ‘filter-group’ as *g2*, and specifying a minimum frequency for *g1* and a maximum frequency for *g2* in the filtering. These types of group-based filterings can be cumbersome and computationally very demanding for other types of analysis strategies, but with the *canvasDB* it can be executed using one single R command.

### Performance of canvasDB filtering analyses

**Figure 4.**
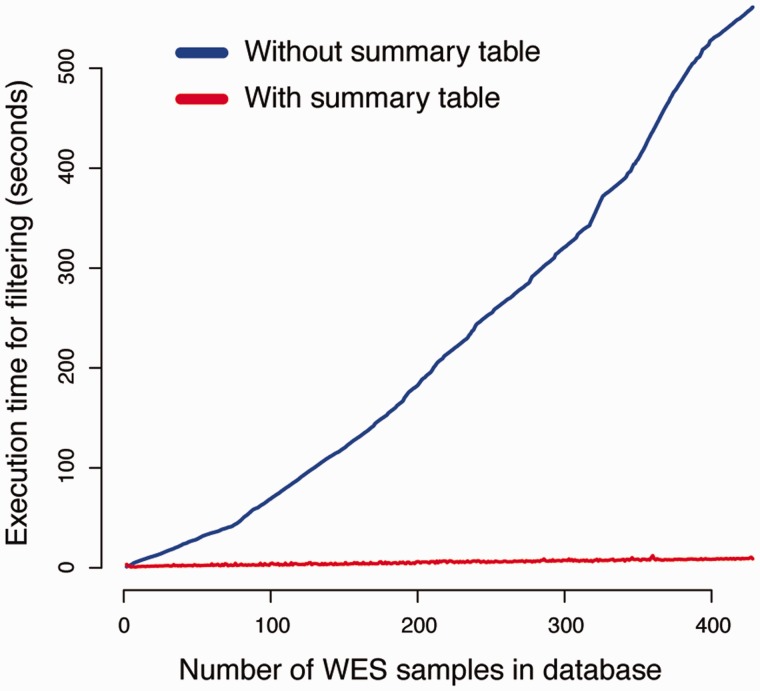
Summary tables speed up the variant filtering. The graphs show the execution times for a simple filtering query (y-axis) as a function of the number of WES samples in the database (x-axis). The task was to detect all variants that were shared by two individuals in the WES database, while absent from all other individuals. The blue line shows the performance of a naïve method that does not use the summary tables for filtering. When summary tables are used the execution time can be dramatically reduced, at least for larger database sizes, as indicated by the red line.

To evaluate the efficiency of using summary tables on filtering performance, we compared the execution time of our *canvasDB* filtering implementation with a simplistic method where variants are analyzed from separate database tables (see Methods for implementation details). For this comparison, we measured the execution time of a simple filtering query as a function of the size of the database. The task was to detect SNPs that exclusively occur in two randomly selected individuals from our WES database (see Figure 2 and Methods). As seen in Figure 4, the execution time for the simplistic method increases linearly with the size of the database, and the analyses take >500 s when the database contains >400 samples. In contrast, the performance of our strategy with summary tables is independent of the number of samples in database, and all of these filtering analyses are completed within 15 s. The scalability of the filtering function implies that a there is essentially no limitation of the number of samples that can be analyzed in the system.

**Figure 5.**
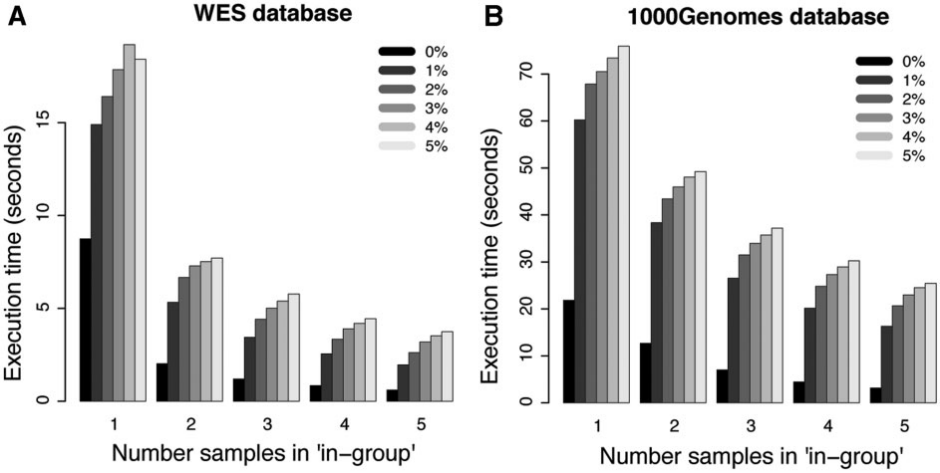
Filtering performance for WES and WGS datasets. Each bar in the plots shows the average execution time for 10 filterings with randomly selected individuals in the ‘in-group’. The five different groups of bars in each of the panels show the results when 1, 2, 3, 4 and 5 individuals are present in the ‘in-group’, respectively. The different shades of gray corresponds to results where at most 0%, 1%, 2%, 3%, 4% and 5% of the individuals in the ‘filter-group’ were allowed to carry the variant. (**A**) Results for filterings in the WES database. (**B**) Results for filterings in the 1000 Genomes WGS database.

For more complicated filtering analyses, such as the ones illustrated in Figure 3D and E, the simplistic method without the use of summary tables is no longer practically feasible. However, the *canvasDB* filtering is very powerful also in these situations. In Figure 5A, we show execution times for WES analyses where we are looking for potentially damaging SNP mutations (i.e. nonsynonymous, stop-gain, stop-loss or splice site) that are shared between 1, 2, 3, 4 and 5 individuals while they may be present in the ‘filter-group’ at frequencies ranging from 0% up to 5%. In all these cases, the analysis was complete within 20 s. Moreover, and perhaps a little counter-intuitive, the filtering becomes more rapid as the number of samples in the ‘in-group’ increases from 1 to 5. The likely explanation for this behavior is that the number of shared candidate variants decreases as the ‘in-group’ grows, thereby speeding up the analysis. Another important observation is that the execution time increases only marginally when allowing higher frequencies of samples in the ‘filter-group’ to carry the mutation. We also performed a similar analysis for the WGS data from the 1000 Genomes project, and in this case the execution times were at most 80 s (see Figure 5B). These filtering results for the 1000Genomes project data show that our system is capable of detecting candidate disease causing mutations from large-scale WGS projects in just a matter of seconds.

### Detection of a disease-causing mutation

A recent example that illustrates the usefulness of *canvasDB* for a WES project was the identification of the causative mutation for Welander Distal Myopathy (WDM) (25). WDM is an autosomal dominant disorder characterized by distal limb weakness, which was previously associated by linkage analysis to a region on chromosome 2 (26). To identify the causative variant, WES was performed on two unrelated individuals, using SOLiD5500 sequencing, and the variant calling results were imported in *canvasDB*. The analysis was then performed as illustrated in Figure 3C, having the two affected individuals in the ‘in-group’. All other samples in the database were assigned to the ‘filter-group’, and we allowed at most one of the ‘filter-group’ samples to carry the mutation.

The result of the analysis was a list of candidate variants that satisfied the filtering conditions. A total of 13 SNP- and 2 indel-candidates were found, and the SNPs obtained in this filtering are shown in Table 1. Of these, 2 SNPs were identified in the linkage region on chromosome 2, located in *ARHGAP25* and *TIA1*, both of them heterozygous and nonsynonymous, and therefore strong candidates for causing WDM. Experimental validation confirmed that *TIA1* was the causative mutation for WDM (25, 27). This example demonstrate that *canvasDB* is a very efficient tool for clinical sequencing, and as illustrated by Figure 3, it can be applied to many other types of genetic analyses. Moreover, the system can handle large numbers of samples and becomes more powerful as the database grows, as a large ‘filter-group’ increases the efficiency of the filtering analyses in removing polymorphisms and technical artifacts specific to individual sequencing technologies.

**Table 1.** Candidate SNP variants for Welander Distal Myopathy (WDM)

Pos	Snp137	Class	Gene
chr1:16535487	rs143314517	Nonsynonymous	*ARHGEF19*
chr2:69049697	rs199643431	Nonsynonymous	*ARHGAP25*
chr2:70439862		Nonsynonymous	*TIA1*
chr3:10082028	rs186545410	Intronic	*FANCD2*
chr5:114916295	rs201468090	Nonsynonymous	*TICAM2*
chr7:70240520	rs145296947	Intronic	*AUTS2*
chr8:113655825		Intronic	*CSMD3*
chr10:24833705	rs1888656	Intronic	*KIAA1217*
chr19:1356844	rs118122389	Intronic	*MUM1*
chr19:1370741	rs199763366	Synonymous	*MUM1*
chr19:32131143		Intergenic	
chr22:45937910		Intronic	*FBLN1*
chrX:68424972		Intergenic	

### Re-analyzing data from the 1000 genomes project

Once the data from the 1000 Genomes Project has been stored in a local *canvasDB* installation, it can be re- analyzed in many different ways. We first attempted to extract population-specific variants for each of the 14 populations. For these analyses, we performed filterings as outlined in Figure 3E, requiring the variant to be present in at least 10% of the individuals in the population of interest (corresponding to group *g1* in Figure 3E) and at most 1% among all other individuals (group *g2*). A summary of the results and execution times is shown in Table 2. The time required for these group-based filterings is <1 h for most of the populations, with a few exceptions that take up to around 20 h. This variation was directly correlated to the number of detected population-specific variants, so that the filterings resulting in the highest number of candidate SNPs (e.g. for LWK, YRI and IBS) also required the longest execution time (Table 2). Genome-wide screenings for genetic differences between large groups of samples are computationally very demanding, and the fact that data from the 1000 Genomes Project can be re-analyzed in a matter of hours demonstrates that *canvasDB* is scalable to whole genomes and has a very powerful filtering tool.

**Table 2.** Results of population-based filtering on 1000Genomes WGS data

Population	Population-specific SNPs^a^				Execution time
	Stopgain	Nonsyn.	Splicing	Other	
GBR	0	10	0	1328	30 m
FIN	5	116	1	20 425	40 m
CHS	2	105	1	19 979	40 m
PUR	0	25	0	6674	39 m
CLM	0	72	0	13 019	43 m
IBS	4	262	1	45 648	4 h 17 m
CEU	0	4	0	842	58 m
YRI	14	1118	14	296 746	13 h 26 m
CHB	0	58	0	10 302	33 m
JPT	3	260	1	43 658	59 m
LWK	12	1256	7	358 884	20 h 25 m
ASW	1	133	1	35 914	49 m
MXL	6	223	4	39 226	59 m
TSI	0	3	1	1620	29 m

^a^Population-specific SNPs are required to be present in at least 10% of the individuals in the population, and at most 1% of all other individuals.

**Figure 6.**
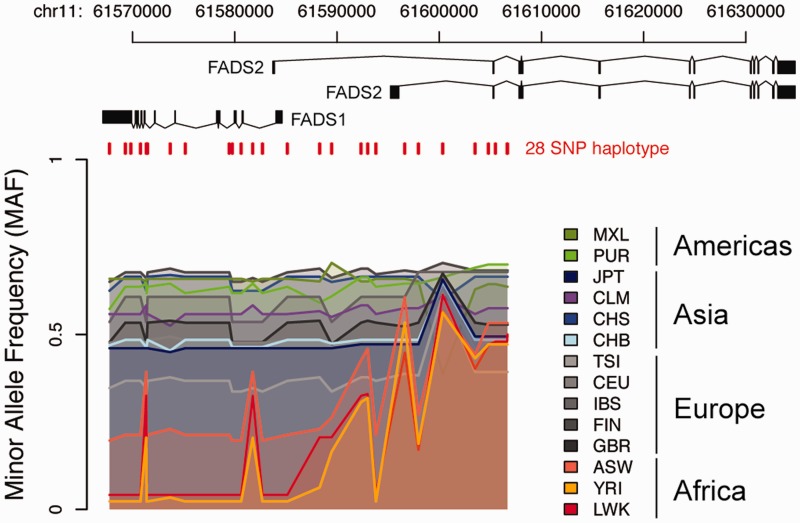
Visualization of 10 000 Genomes data in a specific region. The colored lines show the Minor Allele Frequencies (MAF) in different 1000 Genomes populations for 28 SNPs on a haplotype, denoted *haplotype D*, over the FADS region on chromosome 11. At the top are the transcript isoforms of *FADS1* and *FADS2*. Red vertical lines mark the genomic positions of the 28 SNPs on haplotype *D*. Haplotype frequency varies between populations having ancestry on different continents, with lowest MAF seen in populations with African ancestry and highest MAF in American populations.

It is straightforward to add on new functionality for analyzing data stored in *canvasDB*, and here we give an example how the 1000 Genomes data can be used to investigate a specific genomic region. The region encompasses the *FADS1* and *FADS2* genes, where we previously detected a human specific haplotype (haplotype *D*) that leads to increased levels of omega-3 and omega-6 polyunsaturated fatty-acids (28). By writing a function in R that makes use of the biomaRt Bioconductor package (29) as well as built in functions from R, we were able to extract positions for genomic features, to combine those with the 1000 Genomes variants from the database, and to produce a plot to visualize the data across the region (see Figure 6). Consistent with our previous observations, haplotype *D* is more common in the African populations compared with other continents (28). This example for the FADS region demonstrates how the *canvasDB* can be used in conjunction with R/Bioconductor to annotate and visualize the genetic variant calls stored in the database. However, this is just one very specific example and there are countless other types of analyses that could be performed using the wide range of bioinformatics tools and packages available from R/Biocounductor.

## Discussion

*CanvasDB* is a software designed to handle extremely large sets of genetic variant data. In comparison with many freely available alternatives (30, 31), and commercial solutions, *canvasDB* has a unique capacity for storing variants from large-scale whole genome projects in a single database and has integrated the functionality to perform customized filtering analyses. The recently published the GEMINI framework (32) has similar aims and functions as *canvasDB*. However, one distinctive difference between the two systems is that GEMINI loads a single VCF containing multiple samples into a database and currently lacks the functionality to load additional samples into an existing database. Thus GEMINI is suited mainly for projects consisting of a fixed number of samples that can be collected and summarized into a single VCF file, while other projects where new samples are being continuously added to the existing database are more difficult to handle in this way. *CanvasDB* is on the other hand specifically designed for recalculating the summary tables as new samples are added. In developing *canvasDB*, we have focused on optimizing the performance and functionality for users having some basic computational background, rather than developing graphical user interfaces to make the system accessible to users from the bio/medical field. We believe that for those who know how to execute simple commands in R, *canvasDB* will considerably reduce the time for analysis compared with most other systems. The integration with R also makes it possible to use external R/Bioconductor packages (14, 24), or to easily add custom functions or plug-ins for further analyses.

It is possible to set up the database on a central server and store data from a large number of research groups, but in its present configuration *canvasDB* does not support users to log in and access only their own data. Therefore, different research groups might prefer to have their own installation, giving them complete control and exclusive access to their data. Having the data stored locally also avoids any debate concerning ethical issues or integrity related questions that could arise from transferring and analyzing sensitive genetic information from human subjects on external servers or ‘clouds’.

Theoretically, there is no limit to the number of samples or variants that can be imported into *canvasDB*. However, extremely large datasets require hardware with sufficient RAM and disk space. Moreover, MySQL may need to be re-configured as the number of samples and variants in the database increase. Here we have demonstrated the scalability of *canvasDB* by importing >4.4 billion variants from WGS of 1092 samples. There is no technical reason why it should not be possible to host tens of thousands of samples within the system, at least for WES experiments, which normally gives output files with a size about 1–2% as compared with WGS.

*CanvasDB* is a general software framework that can be used for other types of human sequencing experiments than those described in this manuscript, and also for nonhuman species. Data from any sample can be imported, as long as the variants in the database have been detected by mapping against a well-defined reference sequence. This implies that a similar database system could be generated for any organism with a known reference sequence. Another application of *canvasDB* is as a storage system of the results from clinical sequencing of specific gene panels. This would generate a local database for clinical research and for evaluation of the clinical diagnostic results. It is even possible to import variant calls from RNA-sequencing datasets into the system, together with the variant calls from genomic DNA. Such information combined can be useful for example when studying imprinting, allele- specific expression or RNA editing.

In summary, we believe the *canvasDB* infrastructure is ideal for research groups or institutions in need of an efficient and flexible system for management and analysis of the large-scale genetic data generated by MPS technologies.

## Availability

*CanvasDB* is freely available to personal, academic and nonprofit use only. The software is available from: https://github.com/UppsalaGenomeCenter/CanvasDB

## Supplementary Data

Supplementary data are available at *Database* Online.

## Funding

This work was funded by the Science for Life Laboratory Uppsala and the Swedish Natural Sciences Council grant for National Research Infrastructure entitled National Genomics Infrastructure (NGI), and project grants from the Swedish Natural Sciences council to U.G. Funding for open access charge: Science for Life Laboratory, Uppsala 

*Conflict of interest*. None declared.

## Supplementary Material

Supplementary Data
