# Supplementary material for: CanvasDB: a local database infrastructure for analysis of targeted- and whole genome re-sequencing projects
Source: Database (Oxford). 2014 Oct 3;2014:bau098. doi: 10.1093/database/bau098 (PMC4184106; doi:10.1093/database/bau098)
Supplement: Supplementary Data [file supp_2014_bau098_index.html]

CanvasDB: a local database infrastructure for analysis of targeted- and whole genome re-sequencing projects — Supplementary Data 

# CanvasDB: a local database infrastructure for analysis of targeted- and whole genome re-sequencing projects

## Supplementary Data

files

**Files in this Data Supplement:**

- Supplementary Data - pdf file
