# Supplementary material for: CanvasDB: a local database infrastructure for analysis of targeted- and whole genome re-sequencing projects
Source: Database (Oxford). 2014 Oct 3;2014:bau098. doi: 10.1093/database/bau098 (PMC4184106; doi:10.1093/database/bau098)
Supplement: Supplementary Data [file supp_bau098_Ameur_et_al_supplementary.pdf]

Supplementary Information:

CanvasDB: A local database infrastructure for analysis of targeted- and whole genome re-sequencing projects

Adam Ameur[1], Ignas Bunikis[1], Stefan Enroth[1] and Ulf Gyllensten[1]

[1]Department of Immunology, Genetics and Pathology, Science for Life Laboratory, Uppsala University, Sweden

Supplementary Tables 2
Supplementary Information 4
  Implementation of rapid filtering analyses 4
  CanvasDB file formats 6
    SNP file format...6
    Indel file format..6
  File format for adding new samples to the database 7

Supplementary Tables

Supplementary Table S1. Database structure for the SNP summary table

Column	Type	Comment
**SNP_id**	string	Unique id for each SNP in the database
**chr**	string	Chromosome
**pos**	integer	SNP coordinate on chromosome
**ref**	string	Reference allele
**alt**	string	Alternative allele (SNP allele)
**nr_samples**[a]	integer	Number of samples in database where SNP is present.
**samples**[a]	blob	A binary text string consisting of samples where the SNP is present. Each sample is represented by its sample ID number. This binary text string is a comma separated string of sample ID numbers.
**snp137**[b]	string	rs id from dbSNP (if SNP is present in dbSNP)
**snp137common**[b]	string	rs id from dbSNP common, a database of SNPs present in at least 1% frequency
**class**	string	Classification of SNP based on Annovar (intronic, exonic, nonsynonymous, splicing, synonymous etc)
**severity**	integer	A number from 1 to 5 representing the putative damaging effect of SNP. Variants that are changing the protein structure have high numbers, while intronic and non-coding variants have low numbers.
**gene**	string	Name of gene where SNP is located (if any)
**details**	string	Additional SNP information from Annovar, codon position, amino acid substitution etc.
**sift**	string	SIFT score
**polyphen**	string	PolyPhen score
**phylop**	string	PhyloP score
**lrt**	string	LRT score
**mut_taster**	string	MutTaster score
**gerp**	string	GERP score

[a] The 'nr_samples' and 'samples' fields needs to be updated each time a new sample is added to the database.
[b] In this example the database is using annotations from v137 of dbSNP, this can be updated to later versions.

Supplementary Table S2. Database structure for the indel summary table

Column	Type	Comment
**indel_id**	string	Unique id for each indel in the database
**chr**	string	Chromosome
**start**	integer	Indel start coordinate on chromosome
**end**	integer	Indel end coordinate on chromosome
**ref**	string	Reference allele
**alt**	string	Alternative allele (indel allele)
**type**	string	Indel type, i.e. insertion or deletion
**size**	integer	Size of indel
**nr_samples**[a]	integer	Number of samples in database where SNP is present.
**samples**[a]	blob	A binary text string consisting of samples where the SNP is present. Each sample is represented by its sample ID number. This binary text string is a comma separated string of sample ID numbers.
**snp137**[b]	string	rs id from dbSNP (if indel is present in dbSNP)
**snp137common**[b]	string	rs id from dbSNP common, a database of SNPs present in at least 1% frequency
**class**	string	Classification of indel based on Annovar (intronic, frameshift, splicing, etc)
**severity**	integer	A number from 1 to 5 representing the putative damaging effect of SNP. Variants that are changing the protein structure have high numbers, while intronic and non-coding variants have low numbers.
**gene**	string	Name of gene where SNP is located (if any)
**details**	string	Additional SNP information from Annovar, codon position, amino acid substitution etc.

[a] The 'nr_samples' and 'samples' fields needs to be updated each time a new sample is added to the database.
[b] In this example the database is using annotations from v137 of dbSNP, this can be updated to later versions.

Supplementary Information

Implementation of rapid filtering analyses

The SNP and indel summary tables (see Supplementary Tables S1 and S2) are crucial for the to the rapid variant filtering in the *canvasDB* system. This section describes the main design of the filtering functionality. The filtering itself is performed by two functions in R, `filterSNPs()` and `filterIndels()`, which queries the summary tables. The filtering functions take the following main arguments:

**inSamples**	Group of samples expected to have a shared variant. Corresponds to 'in-group' in Figure 3A.
**filterSamples**	Group of samples where variant should not occur, i.e. negative controls. Defaults to all other samples outside the 'in-group'. Corresponds to 'filter-group' in Figure 3A.
**discardSamples**	Group of samples not used in the filtering. Defaults to an empty set. Corresponds to 'discard-group' in Figure 3A.
**minIn**	Minimum number of individuals in the 'in-group' that are required to have a shared variant. Defaults to all samples in 'in-group'.
**maxOthers**	Maximum number of individuals in the 'filter-group' that are allowed to carry the variant. Defaults to zero.
**minSeverity**	A score between 1-5 representing the potential damaging effect of the variant. Defaults to 3, meaning that only variants with a score of at least 3 are considered, i.e. amino acid sequence altering variants.
**dbSNPfilterCommon**	A flag indicating whether only rare variants (not present in dbSNPCommon) are to be considered. Defaults to FALSE.

When the filtering functions are called with the above arguments, the following steps are executed.

1. Based on the number of samples in the `inSamples`, `filterSamples` and `discardSamples` groups, as well as the arguments `minIn` and `maxOthers`, the two values `minSamples` and `maxSamples` are calculated, representing the minimal and maximal number of samples in which candidate variant should be found. Variants present in fewer samples than `minSamples` or more than `maxSamples` cannot fulfill the filtering criteria.

2. From the SNP or indel summary table only variants where `nr_samples` is in the range between `minSamples` and `maxSamples` are returned. This is done by a MySQL query that can be rapidly executed since the relevant columns of the summary tables are indexed. In the same query, filtering on `minSeverity` and `dbSNPfilterCommon` are performed (if those arguments were set in the function call). The resulting variants from the MySQL query are returned into R. This step can reduce the number of candidate variants dramatically.

3. The variants returned into R from step 2) are present in a number of samples that is consistent with the original filtering function call. For these remaining variants, the sample information is present in the binary text string `samples`. R immediately converts the binary `samples` string into a vector object, resulting in a numerical vector with sample IDs for each of the candidate variants. These vectors are then compared to the samples in the `inSamples` group using built-in vector matching functions in R. Those that fulfill the original filtering criteria having at least `minIn` samples present in the `inSamples` group represent the final set of candidate variants.

4. Once the candidate variants have been detected from the summary table (in steps 1-3 above) the actual data for each of the samples (read counts, quality scores etc) are fetched from the individual SNP and indel tables for each of the samples, after which the final results are formatted as a table that is returned to the user.

As described above, the filtering is essentially performed by one single MySQL query on indexed columns in a summary table and subsequent analyses of vector objects in R. This allows for very rapid execution for most types of filtering analyses.

CanvasDB file formats

When importing SNP and indel variant calls into *canvasDB*, the files are parsed into a specific format that can be imported into the database. These file formats for SNPs and indels are described in this section. In the system there are pre-defined functions for parsing some common variant call file formats (e.g. VCF) into the cdb files. However, it is also possible to parse other formats into cdb files. One advantage of parsing the files outside of the system is that it makes the import into the database faster.

SNP file format

A separate SNP file needs to be created for each individual sample. The *canvasDB* SNP file format is a tab delimited text file. Each row corresponds to a SNP and the following columns are required:

**SNP_id**	A unique ID for each SNP in the database. On the format 'chr|pos|ref|alt', e.g. chr1|879482|G|C
**Chr**	Chromosome, e.g. chr1
**Pos**	SNP position on chromosome, e.g. 879482
**Ref**	Reference allele, e.g. G
**Alt**	Alternative allele, e.g. C
**Cov**	Read coverage
**ref_reads**	Number of reference reads
**ref_starts**	Number of reference reads with unique starting points
**ref_qual**	Quality score for reference allele
**alt_reads**	Number of alternative reads
**alt_starts**	Number of alternative reads with unique starting points
**alt_qual**	Quality score for alternative allele
**Het**	Heterozygosity flag. 0=homozygous, 1=heterozygous

Indel file format

The file format for indels is a tab delimited text file, similar to the SNP file format described above. Each row corresponds to an indel and the following columns are required:

**indel_id**	A unique ID for each indel in the database. On the format 'chr|start|end|ref|alt', e.g. chr1|1276972|1276973|-|CACA
**chr**	Chromosome, e.g. chr1
**start**	Indel start position on chromosome, e.g. 1276972
**end**	Indel end position on chromosome, e.g. 1276973
**ref**	Reference allele, e.g. -
**alt**	Alternative allele, e.g. CACA (insertion)
**cov**	Read coverage
**ref_reads**	Number of reference reads
**alt_reads**	Number of alternative reads
**het**	Heterozygosity flag. 0=homozygous, 1=heterozygous

File format for adding new samples to the database

The easiest way to add new samples into the database is to construct a text file holding information about multiple samples and send this file as a parameter to the the batchImport() function in R. This section describes the structure of the sample text file.

The sample file is a text file delimited by the character '|'. This implies that the '|' character may not be used in sample names, descriptions or any other fields included in the file. The file should include a header with the following fields:

**canvasId**	Sample identifier used within the *canvasDB* system. Each sequenced sample must have a unique canvasId. These ids are used in filtering analysis functions etc.
**sampleName**	Alternative name of the sequenced samples.
**seq.platform**	Sequencing technology used for the experiments
**library.type**	The library type used for the experiments, for example *WholeExome* or *WholeGenome*
**read.type**	Type of read and read length, for example Frag75, PE100x100 or similar
**capture.method**	Method used for capturing genomic target region
**date**	Date of the sequencing experiment
**principal.investigator**	Name/initials of principal investigator
**instrument.name**	Name of sequencing facility/instrument
**gender**	Gender of sequenced individual
**comments**	Any comments about experiment
**geographic.location**	Geographic origin of sample
**phenotypes**	Text string describing the phenotye
**SNP.file**	Path to SNP data file
**indel.file**	Path to indel data file
**file.format**	File format for SNP/indel files. Currently the following options are supported: *CanvasDB*, *GATK* (VCF), *Lifescope*, *TorrentSuite* (VCF)
**reads.total**	Total number of reads for the sample
**reads.on.target**	Number of reads mapping to target region
